# Population and life-stage specific sensitivities to temperature and salinity stress in barnacles

**DOI:** 10.1038/srep32263

**Published:** 2016-09-01

**Authors:** Ali Nasrolahi, Jonathan Havenhand, Anna-Lisa Wrange, Christian Pansch

**Affiliations:** 1 Department of Marine Ecology, GEOMAR Helmholtz Centre for Ocean Research Kiel, 24105 Kiel, Germany; 2 Department of Marine Sciences – Tjärnö, University of Gothenburg, Tjärnö, 45296 Strömstad, Sweden

## Abstract

Temperature and salinity shape the distribution and genetic structure of marine communities. Future warming and freshening will exert an additional stress to coastal marine systems. The extent to which organisms respond to these shifts will, however, be mediated by the tolerances of all life-stages and populations of species and their potential to adapt. We investigated nauplius and cypris larvae of the barnacle *Balanus (Amphibalanus*) *improvisus* from the Swedish west coast with respect to temperature (12, 20, and 28 °C) and salinity (5, 15, and 30) tolerances. Warming accelerated larval development and increased overall survival and subsequent settlement success. Nauplii developed and metamorphosed best at intermediate salinity. This was also observed in cypris larvae when the preceding nauplii stages had been reared at a salinity of 30. Direct comparisons of the present findings with those on a population from the more brackish Baltic Sea demonstrate contrasting patterns. We conclude that i) *B. improvisus* larvae within the Baltic region will be favoured by near-future seawater warming and freshening, that ii) salinity tolerances of larvae from the two different populations reflect salinities in their native habitats, but are nonetheless suboptimal and that iii) this species is generally highly plastic with regard to salinity.

In the course of global climate change, both means and variances of key abiotic variables, such as temperature, ocean pH and salinity, are projected to shift^1^,^2^,^3^,^4^. These changes are likely to impose strong selective pressures on marine species, and might lead to the evolution of new adaptations and/or increased plasticity (the ability of a single genotype to alter its phenotype in response to temporal or spatial fluctuations in the environment)^5^. Local adaptation and phenotypic plasticity determine the responses of populations to changes in their environment^6^. Thus, multi-population comparisons are urgently needed to better predict pending ecosystem changes.

The Baltic Sea is one of the world’s largest semi-enclosed brackish seas. The strong salinity gradient from the North Sea and Skagerrak, through the Kattegat to the low salinity Baltic Sea drives the distribution of species^7^,^8^ as well as the genetic structure of populations^9^. In the Baltic region, temperature and salinity are projected to undergo stronger shifts (temperature increase of 2 to 6 °C and salinity decrease by up to 50% by 2100) than is projected for other regions worldwide^2^,^3^,^10^,^11^,^12^,^13^, exposing organisms to new combinations of potentially stressful conditions^14^ with distribution shifts of species and potential consequences on an ecosystem level.

Early life-history stages are often considered to be more susceptible to environmental stress than adults^14^,^15^, and may also have a strong influence on the fitness of the subsequent stages^16^. In order to make meaningful projections on population and ecosystem shifts, it is vital to understand the stress impacts on the entire life cycle of an organism.

Intertidal barnacles are ecologically and economically important and widely studied ecosystem engineers (e.g. refs 16,18, 19, 20, 21, 22). Barnacles develop through seven larval stages: six feeding “nauplius” stages, and a non-feeding cypris, highly specialized for settlement^23^, before reaching the benthic adult stage. Energy reserves acquired during the nauplius phase play an important role in subsequent settlement and metamorphosis^24^,^25^.

The euryhaline bay barnacle *Balanus (Amphibalanus*) *improvisus*^26^,^27^ is found in shallow, tidal or non-tidal environments worldwide, at salinities from <1 to 35^28^. *B. improvisus* invaded many brackish habitats worldwide^30^ and can be the dominant calcifier of benthic hardbottom communities. This species can also colonize fully marine environments – albeit with reduced dominance (pers. obs.). In the Baltic Sea, *B. improvisus* is by far the most common barnacle species^31^,^32^. Previous studies have shown that adult *B. improvisus* display strong phenotypic plasticity with respect to salinity^30^.

In the present study, we assessed the tolerance norms of nauplius and cypris stages of the barnacle *B. improvisus* to a range of temperature and salinity levels that occur naturally within the distribution range of *B. improvisus*^28^,^29^, and that characterize present day and future habitats. We studied a population from the Swedish west coast (Tjärnö, Skagerrak; ambient mean salinity ~25) that experiences comparable summer temperature but largely differing salinity regimes than a *B. improvisus* population from the brackish western Baltic Sea (Kiel, Germany, ambient salinity ~15) that has previously been investigated^33^. The present study was designed to be identical to the study by ref. 33, since comparisons of responses in different populations to climate change are crucial for our understanding of the likely success of a species in the near future^34^.

## Results

**Figure 1:**
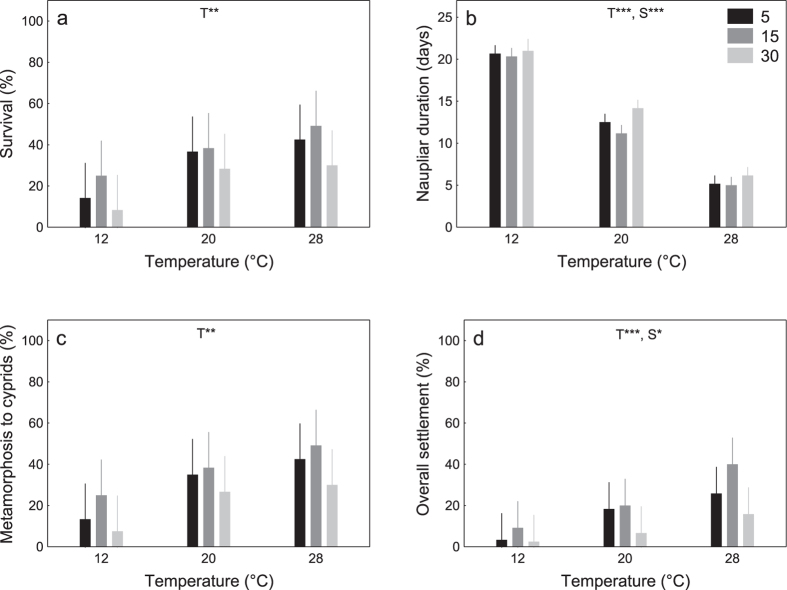
Experiment I. Effect of temperature (T) and salinity (S) on survival (**a**), naupliar duration (**b**), metamorphosis to cyprids (**c**) and overall settlement (**d**) from 20 initial *Balanus improvisus* nauplius larvae per replicate of Experiment I (means ± 95% CI; N = 6). The statistical significance of treatment effects is indicated by *p < 0.05, **p < 0.01, ***p < 0.001 (no significant interactions were observed, Table 1). Posthoc comparisons are illustrated in Table 3 (PERMANOVA pair-wise tests at p < 0.05).

**Figure 2:**
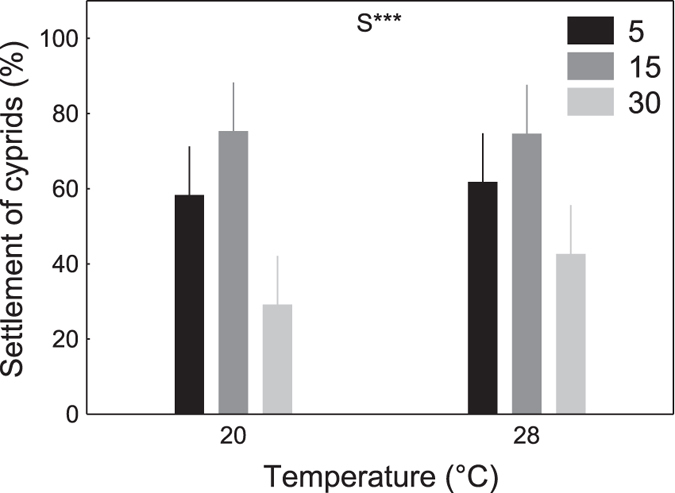
Experiment I - settlement of cyprids. Effect of temperature (T) and salinity (S) on settlement of cyprids from 20 initial *Balanus improvisus* nauplius larvae per replicate of Experiment I (means ± 95% CI; N = 6). The statistical significance of treatment effects is indicated by *p < 0.05, **p < 0.01, ***p < 0.001 (no significant interactions were observed, Table 1). Posthoc comparisons are illustrated in Table 3 (PERMANOVA pair-wise tests at p < 0.05).

We found no statistically significant interactive effects of temperature and salinity on any of the metrics of naupliar performance (Experiment I; Table 1, Fig. 1). However, temperature significantly affected survival, naupliar duration, metamorphosis to cyprids, and overall settlement of *B. improvisus* larvae (Tables 1 and 2, Fig. 1). Warming increased survival (Fig. 1a), accelerated naupliar development and metamorphosis to cyprids (Fig. 1b,c), and increased the overall settlement of larvae (Table 3, Fig. 1d). Naupliar durations were longest at 12 °C and shortest at 28 °C (21 and 6 days, respectively) regardless of salinity (Table 3, Fig. 1b). As mentioned previously, very few larvae survived to settlement in 12 °C (in all salinities) and therefore data from these treatments (Fig. 1) were excluded from the analyses of the settlement of cyprids (Table 1; Fig. 2). After removal of these data, temperature had no statistically significant effect on settlement of cyprids (Table 1, Fig. 2). Salinity, on the other hand, had a significant effect on development (Table 1; Fig. 1b): naupliar duration was longest at salinity 30, regardless of temperature (Table 3, Fig. 1b). Survival, successful metamorphosis to cyprids, and overall settlement of *B. improvisus* larvae were all greatest at intermediate salinity (15) and lowest at highest salinity (30; Table 3, Fig. 1a,c,d). Salinity significantly affected settlement of cyprids (Table 1; Fig. 2): with highest settlement of cyprids at intermediate salinity (15) and lowest settlement at highest salinity (30; Table 3, Fig. 2).

**Figure 3:**
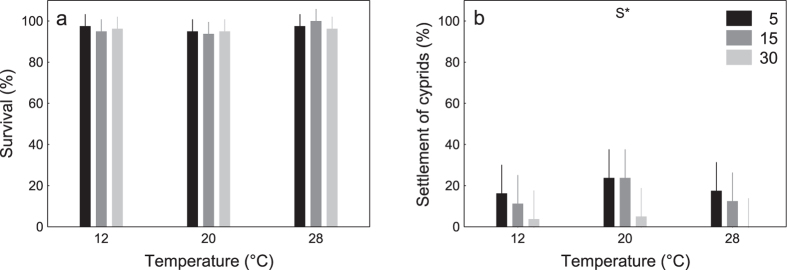
Experiment II. Effect of temperature (T) and salinity (S) on survival (**a**) and settlement of cyprids (**b**) from 10 initial *Balanus improvisus* cyprids per replicate Experiment II (means ± 95% CI; N = 8). The significance of effects is indicated by *p < 0.05, **p < 0.01, ***p < 0.001 (no significant interactions were observed, Table 2). Posthoc comparisons are given in Table 3 (PERMANOVA pair-wise tests at p < 0.05).

For larvae raised in standard culture conditions (salinity of 30, 25 °C) until the cypris stage (Experiment II), almost all (>95%) cyprids survived the 26 d experimental period, regardless of treatment, and there were no significant effects of temperature, salinity, or their combination on survival (Table 2; Fig. 3a). Settlement of cyprids ranged from 5 to 80%, as also demonstrated in other studies of this species^33^,^35^,^36^,^37^,^38^,^39^. Settlement was significantly influenced by salinity (but not temperature; Table 2; Fig. 3b), with settlement being lowest at high salinity (30) – the salinity at which these larvae were cultured (Table 3, Fig. 3b).

**Figure 4:**
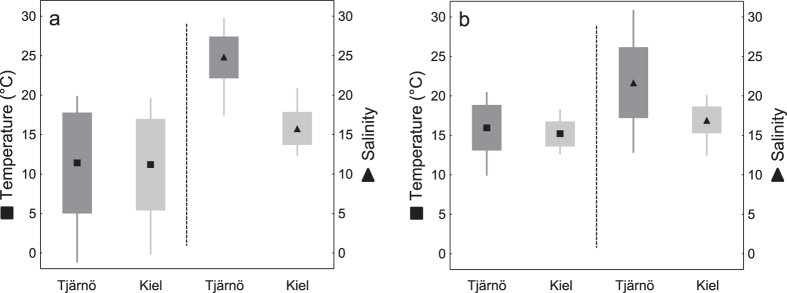
Field data. Field temperature (left, squares) and salinity (right, triangles) in the Tjärnö Archipelago (dark grey) and at the inner Kiel Fjord (light grey) from monthly (2009 to 2013; (**a**) and daily (August to October 2011; (**b**) measurements (±SD (boxes) and minimum to maximum values (lines)).

**Figure 5:**
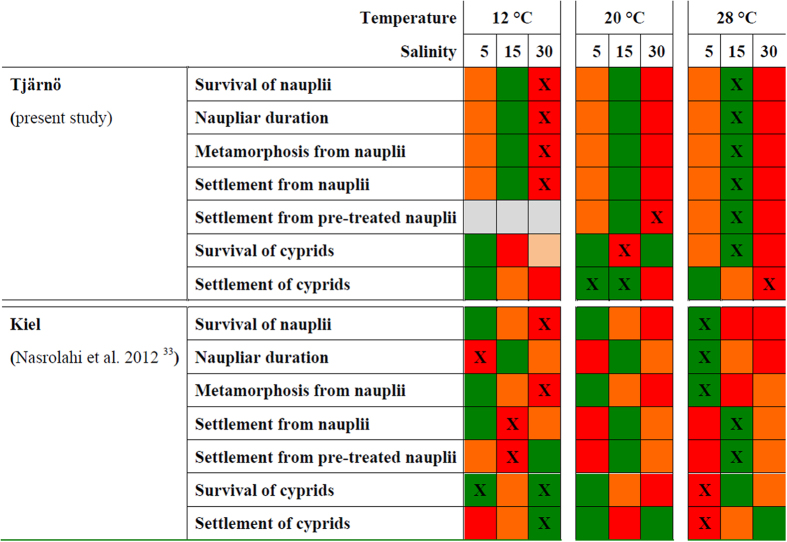
Population comparisons. Direct comparisons of the present results to those from an earlier identical study on a different population^33^ of barnacles at 12, 20 and 28 °C and a salinity of 5, 15 and 30. Best, medium and weak mean performances within a given temperature treatment are indicated by green, orange and red, respectively. An “x” indicates the overall best (green/cross) and weakest (red/cross) performance across all temperatures and salinities. Grey treatment combinations were not tested.

Comparisons of temperature and salinity variability of the Tjärnö Archipelago and the Kiel Fjord show significant differences in mean salinity (ANOVA; 2009 to 2011: F = 350.3, p < 0.001; daily 2011: F = 45.7, p < 0.001) as well as in the fluctuations around means, both being higher at the Tjärnö Archipelago. Temperature regimes did not differ significantly (Fig. 4a,b). Direct comparisons of the present results to those obtained from a similar study by Nasrolahi *et al.*^33^ are given in Fig. 5.

## Discussion

The early nauplius larval stage of *B. improvisus* from Tjärnö followed a stringent pattern of best performance at salinities of 15 and poorest performance at salinity 30 (Figs 1, 2 and 3). The later cyprid stage showed a similar response to salinity with best performances at the lower salinities (5 and 15; Figs 1, 2 and 3). Warmer temperatures generally increased the performance of both, nauplii and cyprids with no threshold being imposed by very warm temperatures of 28 °C (Figs 1, 2 and 3). Thus, both, warming and freshening of surface seawater benefited the larval stages of this *B. improvisus* population from western Sweden. In contrast to the present findings, nauplius larvae from Kiel responded more variable to salinity than nauplii from Tjärnö, but in average, performed best in lower salinity (5 to 15; Fig. 5 and ref. 33). Cyprids in response to the salinity treatments showed best performance at lowest (5) and highest (30) salinities (Fig. 5 and ref. 33).

The inverse relationship between naupliar duration and increasing temperature observed herein (Fig. 1b) is in accordance with previous studies on temperature effects on *B. improvisus*^33^,^40^ as well as on other barnacle species (e.g. *Balanus (Amphibalanus*) *amphitrite*^18^,^19^,41, *B. trigonus*^21^, *B. eburneus*^42^, *Elminius modestus*^43^,^44^ and *Semibalanus balanoides*^43^. This may be a direct consequence of increased metabolic rates^45^, better energy assimilation^41^, and/or an indirect consequence of reduced feeding efficiency and filtration rates at colder temperatures^46^,^47^,^48^. Comparable – albeit less pronounced – patterns of temperature dependency were also observed in the other response variables on nauplii (Fig. 1). Increasing temperature, thus, enables nauplius larvae to develop faster and sooner reach size refuge from predation^49^. There were no strong effects of temperature on cypris mortality and settlement (Figs 2 and 3). There were no interactive effects of temperature and salinity on any of the metrics of naupliar performance (Figs 1, 2 and 3).

Naupliar performance was generally greatest at lower than natural salinities (15; Figs 1 and 4). For larvae that were grown in the treatments from the outset (Experiment I) and when nauplii were raised at 25 °C and a salinity of 30 (Experiment II), cypris settlement was poorest at salinities of 30 (Figs 1, 2 and 3) and best at 15 (Figs 1 and 2) and 5 (Fig. 3), respectively. This, and the fact that all nauplius larvae for the experiments were taken from parents, which had been kept in artificial rearing conditions at high salinities (30), suggests little maternal effects from *B. improvisus* adults to their offspring^50^, and are perhaps more surprising given that in their natural habitat, this population of barnacles routinely experiences higher salinities, closer to those of the highest salinity treatment of 30. These results also contrast markedly with other barnacle species such as *Elminius modestus*^43^ and the closely related barnacle *B. amphitrite*^18^, which also tolerate a relatively broad range of salinities but perform best in fully marine conditions.

It is crucial that projections of future ecosystem changes are based on studies including multiple stressors, various life-history stages, and different populations of a species^34^. Although our present study and the previous study^33^ were not performed simultaneously (raising the possibility of confounding effects due to different times and study-locations) the experimental design of both studies was identical, which facilitates comparison. Interestingly, nauplii from Tjärnö (ambient salinity ~25; Fig. 4) and from Kiel (ambient salinity ~15; Fig. 4) consistently performed best in treatments ~10 salinity units lower than that in their native habitat (i.e. maximum performance was observed in a salinity of 15 and 5 in Tjärnö and Kiel, respectively; Fig. 5 and ref. 33). Moreover, cypris settlement was poorest at a salinity of 30 (Figs 1, 2 and 3; Fig. 5 and ref. 33). Together, these results suggest that larvae from these populations are not well adapted to local salinities, and are indeed maladapted to fully marine conditions. The results from both studies indicate that *B. improvisus* can be considered a true “brackish” species, albeit with a broad salinity tolerance, which has been suggested previously^30^,^51^. Similar responses to temperature in the two populations (Figs 1, 2 and 3 and ref. 33) are perhaps to be expected given the similarity of temperature regimes in the two habitats (Fig. 4).

The population-specific differences of *B. improvisus* larvae in response to salinity observed herein and in Nasrolahi *et al.*^33^ may be due to genetic differences, and/or long-term physiological acclimation (i.e. slow – perhaps trans-generational – plasticity). However, given the relatively recent introduction of this species into the Baltic Sea (<200 years ago)^30^, a high dispersive capacity mediated by free-swimming larvae^52^,^53^ and anthropogenic transport of adults settled on ship hulls^54^, as well as high plasticity with regard to salinity, these differences are very surprising. Contrasting this, in a common-garden experiment, Wrange *et al.*^30^ found little evidence for local adaptation in juveniles and adults from three different populations of this species (including Tjärnö and Kiel). This clearly indicates the importance of investigating different life-history stages in different populations of a species.

Global change has increased the success of biological invaders^55^. Particularly warming facilitates the invasion of non-native species leading to competitive exclusion of native species^56^,^57^,^58^. Recent investigations have elucidated that intertidal barnacles react to current climatic warming by changing the ranges and/or abundance^59^,^60^. Increased abundances of *Austrominius modestus* in Ireland^61^ and on the rocky island of Helgoland^62^,^63^ may also be attributed to seawater warming. Given the benefit of high temperature for the larvae of *B. improvisus* in this study and the potential of this species in colonizing new habitats^64^, it may be expected that warming in shallow marine habitats and estuaries can enhance fouling invasion of this species.

The performance of barnacle larvae is vital for the recruitment success of barnacle populations^65^,^66^,^67^. Our results show positive effects of warming and freshening on *B. improvisus* larval performance in two different populations of this species (present study and ref. 33). Similar positive effects have also been reported for the post-settlement development and reproduction of this species^30^,^68^. Whether *B. improvisus* will respond to future warming and freshening by maintaining or increasing population size will, however, also depend on how this species responds to the combination of other stressors^3^, and on how its competitors, consumers and parasites are affected by these aspects of climate change. Studies on the impacts of ocean acidification and eutrophication, for example, have shown that this species can withstand wide fluctuations in seawater pH – especially if food is plentiful^40^,^69^,^70^. Based on our results and previous available studies, we suggest that the barnacle *B. improvisus* will benefit from near-future climate changes.

## Conclusions

Available evidence suggests that both larval stages (this study and ref. 33) and juvenile to adult stages^30^,^68^ of the barnacle *Balanus improvisus* are likely to respond positively to the warming and freshening of coastal areas as is projected in near-future climate change models^2^,^3^,^10^,^11^,^12^,^13^. *B. improvisus* displays a broad salinity tolerance – albeit with preference for brackish conditions. High plasticity, in combination with long distance larval dispersal, can be a reason for relatively little local adaptation to the almost fully marine conditions at our study site (Tjärnö, Skagerrak, Sweden). We echo earlier calls^34^,^71^ that an understanding of the responses of marine ecosystems to future change can only be achieved by including multiple simultaneous abiotic stressors, whole life-cycle approaches and life-stage comparisons, and – importantly – direct comparisons of multiple populations of a species.

## Methods

Experiments were conducted at the Sven Lovén Centre for Marine Sciences - Tjärnö, Sweden (58°52.5′N, 11°08.1′E), in October 2009. Newly hatched barnacle nauplii were obtained from routine cultures of broodstock barnacles. Broodstock (several hundred adult *B. improvisus*) were collected in the late August 2009, held in filtered seawater at ~25 °C and salinities of ~30 (salinity was measured using the Practical Salinity Scale) in flow-through seawater, and fed *ad libitum* with freshly hatched *Artemia salina* occasionally supplemented with diatom algae (*Chaetoceros calcitrans*, *Skeletonema marinoi* and *Thalassiosira pseudonana*). Lab conditions were aimed to be a continuation of the field conditions at the time of collection, thus, prolonging summer conditions when barnacles reproduce best. This is done to obtain all year-round availability of nauplii and cyprids, feeding research for well over a decade. Barnacle nauplii were collected on sieves (60 μm mesh) from the seawater outflows of the broodstock tanks. Newly released nauplii were at stage I but developed to stage II within a few hours. Batches of stage-II larvae from different subsets of multiple parents were used for each of two experiments.

Our experimental design followed that of Nasrolahi *et al.*^33^. Briefly, three different temperature (12, 20, 28 °C) and salinity (5, 15, 30; salinity is presented using the Practical Salinity Scale) treatments were applied to barnacle larvae in a fully crossed experimental design using replicate six-well plates (CELL STAR #657160). Especially for the season of larval development, surface temperatures of up to 22 to 24 °C are common in shallow habitats (e.g. 15 year dataset by GEOMAR weather station; Sven Lovén Centre for Marine Infrastructure - Water and weather data), such as the Kiel Fjord or the Tjärnö Archipelago, and will be amplified by future climate shifts (+4 to +6 °C)^2^,^3^,^13^. Temperatures were controlled using thermostatted water baths. Different salinities were obtained by diluting filtered (0.2 μm) seawater (salinity of ~30) with de-ionized water. Target temperatures were maintained at ±0.5 °C (YSI30 Multimeter, Brannum Lane, USA). Each treatment combination was replicated six (Experiment I) or eight (Experiment II) times. To control for “room effects”, the positions of the six-well plates in the water baths were randomly re-distributed every day.

### Experiment I – nauplius to cypris development

Twenty nauplii were incubated in 10 ml filtered (0.2 μm) seawater in each well of the six-well plates under continuous light, and fed daily with a 1:1 mixture of the unicellular algae, *C. calcitrans* and *S. marinoi*, at a concentration of 2 × 10^5^ cells ml^−1^ 21. Every second day, the water in each well was carefully replaced by filtered seawater at the respective temperature and salinity, and fresh food was added. The number of surviving nauplii, cyprids and settled juveniles in each experimental well were observed daily for 21 days using a dissecting microscope (Olympus SZX12). Preliminary trials confirmed that the short handling time of each six-well plate during observations did not cause any significant change in water temperature in any of the treatments.

The resulting data were used to calculate: i) survival (% survivors in form of nauplii, cyprids or settled individuals at the end of the experiment relative to the initial number of nauplii), ii) naupliar duration (days from hatching until 50% of the surviving nauplii had metamorphosed into cyprids), iii) successful metamorphosis to cyprids (% of the initial number of nauplii that metamorphosed into cyprids at the end of the experiment), and iv) overall settlement success (number of settled cyprids at the end of the experiment relative to the initial number of nauplii). We additionally calculated v) settlement of cyprids (number of successfully settled cyprids at the end of the experiment relative to metric iii).

### Experiment II – cypris settlement

Newly released nauplius larvae were held at a density of 0.5 larvae ml^−1^ in filtered (0.2 μm) seawater in 20 l containers at 25 °C and a salinity of 30 (“standard” culture conditions at the time of collection, (see Berntsson *et al.* for culture details see ref. 72)). Containers were gently aerated and provided with a 1:1 mixture of the unicellular algae, *T. pseudonana* and *S. marinoi*, at a concentration of 2 × 10^5^ cells ml^−1^. Every third day, the water in each container was carefully replaced by filtered seawater and fresh food was added. When cyprids appeared in the cultures (usually after 6 to 7 days), the cultures were sieved (200 μm) and cyprids were collected.

Ten cyprids were incubated under continuous light in 10 ml filtered (0.2 μm) seawater in each well of the six-well plates at the temperature and salinity combinations outlined above. The transition from initial to target temperature and salinity conditions was achieved gradually (2 salinity units h^−1^, 1 °C h^−1^) to prevent acute shock to the cyprids. Every second day, the water in each well was carefully replaced with fresh filtered seawater at the respective temperature and salinity. Settlement and survival of cyprids were monitored daily over 26 days. From these data we calculated: i) survival and ii) settlement of cyprids.

### Abiotic seawater conditions in Tjärnö and Kiel

Field temperature and salinity were obtained from measurements at 1 m depth (representative of *B. improvisus* larval- as well as adult-stage habitats) monthly from 2009 to 2013 and daily from August to October 2011. Mean field temperature and salinity were measured at the Tjärnö Archipelago, by the Sven Lovén Centre for Marine Sciences, Sweden (58°52.5′N, 11°08.1′E) using a YSI30 Multimeter (Brannum Lane, USA) and at the inner Kiel Fjord, Germany (54°19.5′N, 10°09.0′E) using a CTD60M (Sea & Sun Technology, Germany) monthly from 2009 to 2013 and daily from August to October 2011 at 1 m depth.

### Statistical analysis

Both experiments consisted of replicated fully factorial designs, with temperature (three levels) and salinity (three levels) as fixed factors. Since very few larvae in 12 °C (at any salinity) survived to settlement in Experiment I, all data from 12 °C treatments were excluded from the analyses. Remaining data were strongly non-normal (Shapiro–Wilk’s W-test), although variances were not heterogeneous (Cochran’s test), and therefore we used permutation-based multivariate analysis of variance based on 9999 permutations and Euclidean distance matrices and post hoc pair-wise comparisons (PERMANOVA + 1.0.2 add-on for PRIMER 6.1.12)^73^,^74^. Salinity and temperature data of the two habitats were tested for normality (Shapiro–Wilk’s W-test) and homogeneity of variances (Levene’s test) and were compared using parametric statistics (ANOVA; STATISTICA 8.0, Stat- Soft, Inc., USA).

### Data availability

All data are available from PANGAEA at: https://doi.pangaea.de/10.1594/PANGAEA.864034.

## Additional Information

**How to cite this article**: Nasrolahi, A. *et al.* Population and life-stage specific sensitivities to temperature and salinity stress in barnacles. *Sci. Rep.* **6**, 32263; doi: 10.1038/srep32263 (2016).

## Figures and Tables

**Table 1:** Experiment I.

		d.f.	SS	MS	Pseudo-F	p	p (MC)
Survival	Temperature (T)	2	5969	2985	6991	0.004	0.003**
	Salinity (S)	2	2119	1060	2482	0.090	0.096
	T × S	4	219,4	54,86	0.129	0.970	0.971
Naupliar duration	Temperature (T)	2	2088	1044	763.8	<0.001	<0.001***
	Salinity (S)	2	23,82	11,91	8713	<0.001	<0.001***
	T × S	4	9407	2352	1721	0.151	0.161
Metamorphosis to cyprids	Temperature (T)	2	6103	3051	6902	0.002	0.003**
	Salinity (S)	2	2344	1172	2651	0.083	0.080
	T × S	4	177,8	44,44	0.101	0.980	0.983
Overall settlement	Temperature (T)	2	4459	2230	8999	0.001	0.001**
	Salinity (S)	2	1951	975,5	3937	0.028	0.026*
	T × S	4	610,2	152,6	0.616	0.653	0.653
Settlement of cyprids (excluding the 12 °C treatment)	Temperature (T)	1	266,8	266,8	0.719	0.400	0.398
	Salinity (S)	2	9336	4668	12,57	<0.001	<0.001***
	T × S	2	318,1	159	0.428	0.653	0.652

Effect of temperature and salinity on survival, naupliar duration, metamorphosis to cyprids, overall settlement and settlement of cyprids (the latter – excluding the 12 °C treatments since no metamorphosed nauplii survived to settlement in this treatment) of *Balanus improvisus*. PERMANOVA, p (MC) = p value after Monte Carlo correction; significance of effects indicated by *p < 0.05, **p < 0.01, ***p < 0.001 (N = 6).

**Table 2:** Experiment II.

		d.f.	SS	MS	Pseudo-F	p	p (MC)
Survival	Temperature (T)	2	133330	66667	0.985	0.393	0.371
	Salinity (S)	2	8333	4167	0.062	0.941	0.943
	T × S	4	83333	20833	0.308	0.881	0.868
Settlement of cyprids	Temperature (T)	2	852780	426390	1104	0.349	0.342
	Salinity (S)	2	3536100	1768100	4577	0.013	0.014*
	T × S	4	272220	68056	0.176	0.948	0.951

Effect of temperature and salinity on survival and settlement of cyprids of *Balanus improvisus*. PERMANOVA, p (MC) = p value after Monte Carlo correction; significance of effects indicated by *p < 0.05, **p < 0.01, ***p < 0.001 (N = 8).

**Table 3:** Post-hoc tests following PERMANOVA statistics of Experiment I and II (Tables 1 and 2).

		Temperature				Salinity			
			Salinity 5	Salinity 15	Salinity 30		12 °C	20 °C	28 °C
E-I	Survival (T**)	12 vs. 20	0.049*	0.238	0.083	5 vs. 15	0.085	0.902	0.657
		12 vs. 28	0.012*	0.081	0.074	5 vs. 30	0.166	0.554	0.389
		20 vs. 28	0.658	0.494	0.913	15 vs. 30	0.019*	0.466	0.248
	Naupliar duration (T***, S***)	12 vs. 20	<0.001***	<0.001***	<0.001***	5 vs. 15	0.293	0.312	0.340
		12 vs. 28	<0.001***	<0.001***	<0.001***	5 vs. 30	0.148	0.236	0.002**
		20 vs. 28	<0.001***	<0.001***	<0.001***	15 vs. 30	0.009**	0.001**	<0.001***
	Metamorphosis to cyprids (T**)	12 vs. 20	0.062	0.245	0.102	5 vs. 15	0.066	0.815	0.660
		12 vs. 28	0.010*	0.088	0.066	5 vs. 30	0.154	0.567	0.390
		20 vs. 28	0.598	0.485	0.819	15 vs. 30	0.015*	0.420	0.245
	Overall settlement (T***, S*)	12 vs. 20	0.125	0.330	0.224	5 vs. 15	0.284	0.903	0.228
		12 vs. 28	0.002**	0.017*	0.074	5 vs. 30	0.694	0.242	0.261
		20 vs. 28	0.485	0.173	0.231	15 vs. 30	0.210	0.218	0.067
	Settlement of cyprids (S***)	12 vs. 20	—	—	—	5 vs. 15	—	0.212	0.093
		12 vs. 28	—	—	—	5 vs. 30	—	0.089	0.059
		20 vs. 28	0.790	0.937	0.306	15 vs. 30	—	0.003**	0.008**
E-II	Survival	12 vs. 20	—	—	—	5 vs. 15	—	—	—
		12 vs. 28	—	—	—	5 vs. 30	—	—	—
		20 vs. 28	—	—	—	15 vs. 30	—	—	—
	Settlement of cyprids (S*)	12 vs. 20	0.573	0.330	0.787	5 vs. 15	0.587	—	0.609
		12 vs. 28	0.907	0.865	0.339	5 vs. 30	0.150	0.110	0.047*
		20 vs. 28	0.656	0.371	0.081	15 vs. 30	0.255	0.123	0.024*

Single comparisons were conducted within temperature or within salinity treatments only (significances of main effects of temperature (T) and salinity (S) and single-comparison effects indicated by *p < 0.05, **p < 0.01, ***p < 0.001; p values after Monte Carlo correction).
